# Bioactive Phenylpropanoid Glycosides from *Tabebuia avellanedae*

**DOI:** 10.3390/molecules18077336

**Published:** 2013-06-24

**Authors:** Maorong Suo, Tomihisa Ohta, Fumihide Takano, Shouwen Jin

**Affiliations:** 1 Department of Pharmacognosy and Chemistry of Natural Products, Faculty of Pharmaceutical Sciences, Kanazawa University, Kakuma-machi, Kanazawa 920-1192, Japan; 2 Tianmu College, Zhejiang Agriculture and Forestry University, Linan 311300, China

**Keywords:** *Tabebuia avellanedae* Lorentz ex Griseb, phenylpropanoid glycosides, antioxidant activity

## Abstract

Three novel phenylpropanoid glycosides **2**, **5**, **6** were isolated from water extract of *Tabebuia avellanedae*, together with three known phenylpropanoid glycosides **1**, **3**, **4**. All compounds were identified on the basis of spectroscopic analysis and chemical methods and, for known compounds, by comparison with published data. All isolated compounds showed strong antioxidant activity in the DPPH assay, and compound **5** give the highest antioxidant activity among all compounds, with an IC_50_ of 0.12 µM. All compounds exhibited moderate inhibitory effect on cytochrome CYP3A4 enzyme.

## 1. Introduction

**Figure 1:**
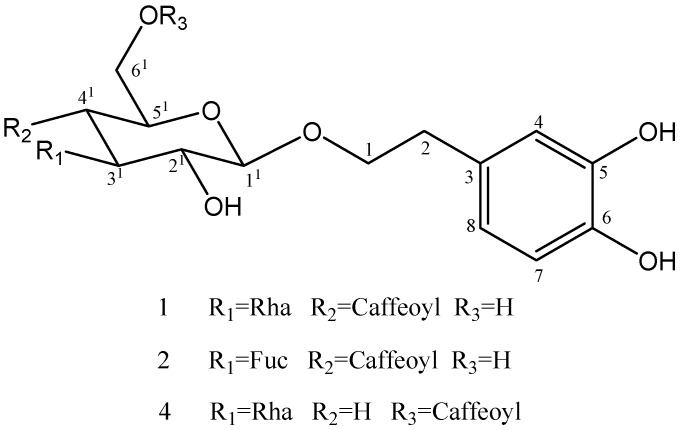
Structure of compounds **1**, **2**, **4**.

**Figure 2:**
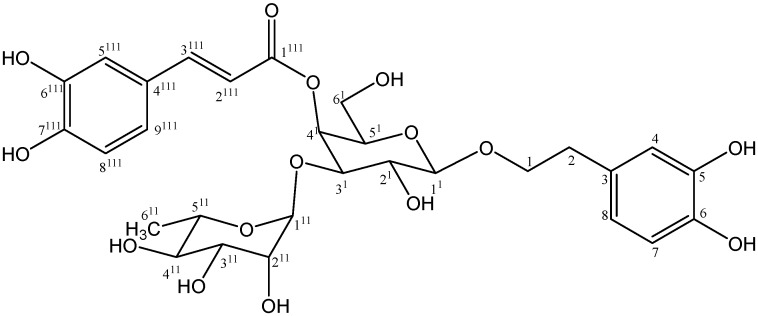
Structure of compound **3**.

**Figure 3:**
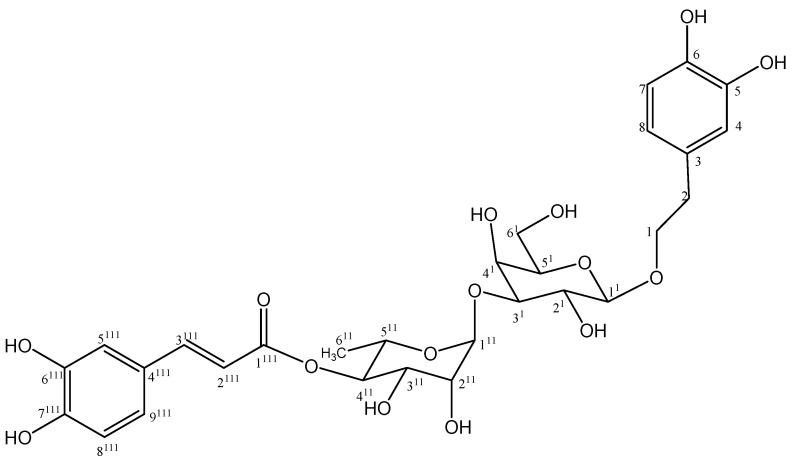
Structure of compound **5**.

**Figure 4:**
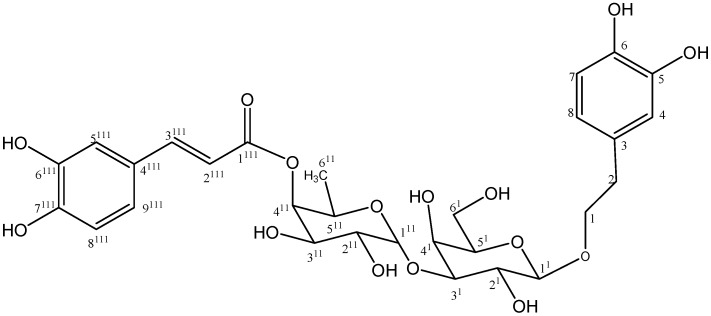
Structure of compound **6**.

*Tabebuia avellanedae* Lorentz ex Griseb is a tree found in tropical rain forests in the northeast of Brazil. Its purple bark, known as “Taheebo”, have been traditionally used for various ethnopharmacological applications. For example, the inner bark has been used as one of the primary medicines by the Callawaya tribe for over 1,000 years, and Taheebo was externally used as a poultice or concentrated tea for treating a variety of skin inflammatory diseases including eczema, psoriasis, and fungal infections, and even skin cancers [1,2,3,4]. In the phytochemical work reported for the species, naphthoquinones, furanonaphthoquinones, anthraquinones, benzoic acid derivatives, benzaldehyde derivatives, iridoids, coumarins and flavonoids were isolated and identified [5,6,7,8,9]. In the course of our research for antioxidants ingredients from Taheebo, six phenylpropanoid glycosides (Figure 1, Figure 2, Figure 3, Figure 4) were obtained from water extract of it. This class of compounds showed antioxidant activities [10,11,12]. The paper describes the isolation and structural determination of these compounds. All compounds were tested for antioxidant activity and inhibitory potency on CYP3A4 enzyme.

## 2. Results and Discussion

Water extract of dried bark of *Tabebuia avellanedae* was successively partitioned into an EtOAc soluble and H_2_O soluble fraction. A combination of a series of flash chromatographic separations and preparative HPLC of the EtOAc soluble fraction afforded six phenylpropanoid glycosides.

### 2.1. Isolation and Chemistry

The structure of compounds **1**, **3** and **4** were determined on the basis of spectral data comparison to literature data [13,14]. Compound **2** was isolated as a colorless powder, and its molecular formular C_29_H_36_O_15_, was determined on the basis of its high resolution FABMS peak at *m/z* 625.2126 [M+H]^+^. The IR spectrum of **2** displayed a carbonyl group absorption band (1,697 cm^−1^). The ^1^H-NMR spectrum of **2** (Table 1) showed two *trans* olefinic protons at *δ* 6.25 and 7.58 (*J* = 15.8 Hz) and two ABX systems [*δ* 7.04 (*J* = 1.8 Hz), 6.78 (*J* = 8.2 Hz) and 6.95 (*J* = 1.8, 8.2 Hz); and *δ* 6.68 (*J* = 2.4 Hz), 6.66 (*J* = 7.9 Hz) and 6.56 (*J* = 2.4, 7.9 Hz)]. The ^13^C-NMR spectrum of **2** (Table 1) showed 29 carbon signals. Analysis of HMQC and DEPT spectrum of **2** revealed two sugar groups, fourteen olefinic carbons, two methylene groups and a carbonyl signal at *δ* 168.3. Long range correlations between signals at *δ* 148.1 (C-3′′′) and *δ* 6.25 (H-2′′′), *δ* 7.04 (H-5′′′), *δ* 6.95 (H-9′′′), as well as the correlation between signals *δ* 168.3 (C-1′′′) and *δ* 7.58 (H-3′′′) were observed in HMBC spectrum of **2**, indicatingthe presence of caffeoyl moiety. In addition, the correlation between signals at *δ* 72.3 (C-1) and *δ* 2.78 (H-2); *δ* 36.6 (C-2) and *δ* 6.68 (H-4), *δ* 6.56 (H-8) were observed in HMBC spectrum of **2**, suggesting the presence of a 3,4-dihydroxyphenyethyl group. The characteristic pattern of ^1^H-NMR and ^13^C-NMR spectrum of **2** is similar to that of **1**, except for sugar moiety. Acid hydrolysis of **2** afforded d-glucose and l-fucose that were identified by HPLC comparisons with sugar standards.

The signals at *δ* 5.34 (H-1′′) correlated with signals at *δ* 79.9 (C-3′) in the HMBC spectrum of **2**, suggesting fucose moieties linked at C-3′ of glucose. Meanwhile, the location of the caffeoyl at C-4′, and phenyethyl group at C-1′ of the glucose was confirmed by the HMBC spectrum, for the long range correlation peaks between signals at *δ* 168.3 (C-1′′′) and *δ* 4.95 (H-4′); *δ* 72.3 (C-1) and *δ* 4.37 (H-1′) were observed in the HMBC spectrum. Thus, compound **2** was deduced to be 1′-O-β-(3, 4-dihydroxyphenyl)-ethyl-4′-O-caffeoyl-α-l-fucopyranosyl-(l-3′)-d-glucopyranoside.

**Table 1:** ^1^H-NMR and ^13^C-NMR data for compound **1**–**3** (CD_3_OD).

No.	1		2		3	
	δ ^1^H	δ ^13^C	δ ^1^H	δ ^13^C	δ ^1^H	δ ^13^C
1	3.91 (1H, m), 4.04 (1H, m)	72.3	3.71 (1H, m), 4.04 (1H, m)	72.3	3.71 (1H, m), 3.96 (1H, m)	72.4
2	2.78 (2H, m)	36.5	2.78 (2H, m)	36.6	2.78 (2H, m)	36.6
3		131.4		131.4		131.2
4	6.68 (1H, d, *J* = 2.4)	117.1	6.68 (1H, d, *J* = 2.4)	117.1	6.68 (1H, d, *J* = 2.4)	117.0
5		146.1		146.1		146.1
6		144.6		144.6		144.7
7	6.66 (1H, d, *J* = 8.2)	116.2	6.66 (1H, d, *J* = 7.9)	116.3	6.66 (1H, d, *J* = 8.3)	116.2
8	6.55 (1H, dd, *J* = 2.4, 8.2)	121.2	6.56 (1H, dd, *J* = 2.4, 7.9)	121.2	6.55 (1H, dd, *J* = 2.4, 8.2)	121.2
1′	4.36 (1H, d, *J* = 7.9)	104.2	4.37 (1H, d, *J* = 7.6)	104.1	4.39 (1H, d, *J* = 7.5)	104.2
2′	3.52 (1H, m)	76.0	3.54 (1H, m)	76.0	3.80 (1H, m)	73.0
3′	3.81 (1H, m)	81.6	3.85 (1H, m)	79.9	3.82 (1H, m)	81.3
4′	4.91 (1H, m)	70.4	4.95 (1H, m)	70.4	4.97 (1H, m)	70.3
5′	3.38 (1H, m)	76.2	3.42 (1H, m)	76.4	3.39 (1H, m)	76.0
6′	3.50 (1H, dd, *J* = 5.5, 9.6), 3.62 (1H, br d *J* = 9.6)	62.3	3.52 (1H, m), 3.63 (1H, br d, *J* = 5.8)	62.3	4.19 (1H, m), 4.21 (1H, m)	63.3
1′′	5.18 (1H, d, *J* = 1.3)	103.0	5.34 (1H, d, *J* = 1.3)	102.0	5.18 (1H, d, *J* = 1.3)	103.1
2′′	3.72 (1H, m)	72.2	3.91 (1H, m)	72.2	3.91 (1H.m)	72.3
3′′	3.57 (1H, m)	72.0	3.70 (1H, m)	69.8	3.56 (1H, m)	72.0
4′′	3.28 (1H, m)	73.7	3.55 (1H, m)	75.9	3.28 (1H, m)	73.7
5′′	3.55 (1H, m)	70.5	3.78 (1H, m)	67.5	3.55 (1H, m)	70.4
6′′	1.09 (3H, d, *J* = 6.2)	18.4	1.02 (3H d, *J* = 6.2)	18.1	1.08 (3H, d, *J* = 6.2)	18.4
1′′′		168.3		168.3		168.0
2′′′	6.26 (1H, d, *J* = 16.2)	114.6	6.25 (1H, d, *J* = 15.8)	114.6	6.26 (1H, d, *J* = 16.2)	114.4
3′′′	7.58 (1H, d, *J* = 16.2)	147.9	7.58 (1H, d, *J* = 15.8)	148.1	7.59 (1H, d, *J* = 16.2)	148.2
4′′′		127.6		127.6		127.7
5′′′	7.04 ( 1H, d, *J* = 1.8 )	115.2	7.04 (1H, d, *J* = 1.8)	115.1	7.04 (1H, d, *J* = 1.8)	115.2
6′′′		146.8		146.8		146.8
7′′′		149.7		149.9		149.8
8′′′	6.77 (1H, d, *J* = 8.2)	116.4	6.78 (1H, d, *J* = 8.2)	116.5	6.77 (1H, d, *J* = 8.2)	116.4
9′′′	6.95 (1H, dd, *J* = 1.8, 8.2)	123.2	6.95 (1H, dd, *J* = 1.8, 8.2)	123.2	6.95 (1H, dd, *J* = 1.8, 8.2)	123.2

Compound **5** was suggested to have the molecular formula C_29_H_36_O_15_ based on a high resolution FABMS peak at *m/z* 625.2135 [M+H]^+^. The ^1^H-NMR and ^13^C-NMR spectrum (Table 2) exhibited similar signals to compound **2**, except that sugar group and the linkage position. Acid hydrolysis of **5** afforded d-galactose and l-rhamnose, identified by comparison with sugar standards by HPLC. The assignment of the data of the two sugars and their linkage position were determined by detailed analysis of ^1^H-NMR, ^13^C-NMR, DEPT, HMBC and HSQC spectrum, and the signal at *δ* 5.23 (H-1′′) correlated with signal at *δ* 83.3 (C-3′) in the HMBC spectrum of **5**, suggesting a rhamnose moiety linked to C-**3**' of galactose. The HMBC spectrum of **5** showed long range correlation between signals at *δ* 168.3 (C-1′′′) and *δ* 5.05 (H-4′′); signals at *δ* 72.3 (C-1) and *δ* 4.31 (H-1′), indicating a caffeoyl residue at C-4′′, and a phenethyl group at the C-1′ of galactose. Thus, compound **5** was established 1'-*O*-β-(3,4-dihydroxyphenyl)-ethyl-[4′′-*O*-caffeoyl-(α-l-rhamnopyranosyl)]-(l-3′)-d-galactopyranoside.

Compound **6** was deduced to have the molecular formula C_29_H_36_O_15_ based on a high resolution FABMS peak at *m/z* 625.2144 [M+H]^+^. The ^1^H and ^13^C-NMR spectra (Table 2) of **6** exhibited similar signals to compound **2**, except for the sugar moieties. Acid hydrolysis of **6** afforded d-galactose and l-fucose, and identified by HPLC comparison with sugar standards.

**Table 2:** ^1^H-NMR and ^13^C-NMR data for compounds **4**–**6** (CD_3_OD).

No.	4		5		6	
	δ ^1^H	δ ^13^C	δ ^1^H	δ ^13^C	δ ^1^H	δ ^13^C
1	3.69 (1H, m), 3.97 (1H, m)	72.3	3.71 (1H, m), 3.93 (1H, m)	72.4	3.69 (1H, m), 3.97 (1H, m)	72.3
2	2.77 (2H, m)	36.6	2.77 (2H, m)	36.6	2.77 (2H, m)	36.6
3		131.3		131.4		131.3
4	6.66 (1H, d, *J* = 2.4)	117.0	6.68 (1H, d, *J* = 2.4)	117.0	6.66 (1H, d, *J* = 2.4)	117.0
5		146.1		146.1		146.1
6		144.6		144.6		144.6
7	6.62 (1H, d, *J* = 8.2)	116.3	6.66 (1H, d, *J* = 8.2)	116.3	6.62 (1H, d, *J* = 8.2)	116.3
8	6.52 (1H, dd, *J* = 2.4, 8.2)	121.2	6.55 (1H, dd, *J* = 2.4, 8.2)	121.2	6.52 (1H, dd, *J* = 2.4, 8.2)	121.2
1′	4.32 (1H, d, *J* = 7.9)	104.3	4.31 (1H, d, *J* = 7.8)	104.4	4.34 (1H, d, *J* = 7.9)	104.3
2′	3.54 (1H, m)	75.4	3.52 (1H, m)	75.4	3.54 (1H, m)	75.5
3′	3.52 (1H, m)	83.9	3.54 (1H, m)	83.3	3.55 (1H, m)	83.1
4′	3.99 (1H, m)	70.3	4.10 (1H, m)	70.2	3.73 (1H, m)	72.2
5′	3.32 (1H, m)	75.7	3.34 (1H, m)	75.8	3.32 (1H, m)	75.9
6′	4.35 (1H, dd, *J* = 5.9, 12.1), 4.49 (1H, dd, *J* = 2.1, 11.7)	64.6	4.32 (1H, m), 4.47 (1H, dd, *J* = 2.1, 11.7)	64.4	4.32 (1H, br d, *J* = 8.5), 4.47 (1H, dd, *J* = 2.3, 11.7)	64.6
1′′	5.17 (1H, d, *J* = 1.8)	102.7	5.23 (1H, d, *J* = 1.4)	102.2	5.25 (1H, d, *J* = 1.8)	102.3
2′′	3.71 (1H, m)	72.2	3.90 (1H, m)	71.1	3.89 (1H, m )	70.0
3′′	3.94 (1H, m)	72.2	3.67 (1H, m)	72.4	3.41 (1H, m)	70.3
4′′	3.38 (1H, m)	73.9	5.05 (1H, m)	75.5	4.89 (1H, m)	76.0
5′′	3.41 (1H, m)	70.0	3.44 (1H, m)	70.0	4.26 (1H, m)	67.4
6′′	1.23 (3H, d, *J* = 6.2)	17.8	1.25 (3H, d, *J* = 7.0)	17.8	1.15 (3H, d, *J* = 5.9)	17.6
1′′′		169.1		168.3		169.1
2′′′	6.28 (1H, d, *J* = 15.8)	114.8	6.26 (1H, d, *J* = 15.8)	114.6	6.28 (1H, d, *J* = 15.8)	114.8
3′′′	7.56 (1H, d, *J* = 15.8)	147.2	7.58 (1H, d, *J* = 15.8)	147.9	7.56 (1H, d, *J* = 15.8)	147.2
4′′′		127.6		127.6		127.6
5′′′	7.03 (1H, d, *J* = 2.0)	115.0	7.04 (1H, d, *J* = 2.0)	115.2	7.03 (1H, d, *J* = 2.0)	115.0
6′′′		146.7		146.8		146.7
7′′′		149.6		149.7		149.6
8′′′	6.62 (1H, d, *J* = 8.2)	116.5	6.77 (1H, d, *J* = 8.2)	116.4	6.62 (1H, d, *J* = 8.2)	116.5
9′′′	6.88 (1H, dd, *J* = 2.0, 8.2)	123.1	6.95 (1H, dd, *J* = 2.0, 8.2)	123.2	6.88 (1H, dd, *J* = 2.0, 8.2)	123.1

The detailed analysis of the ^1^H-NMR, ^13^C-NMR, DEPT, HMBC and HSQC spectra gave the assignments of the two sugars and their linkage position, and the signals at *δ* 5.25 (H-1′′) correlated with the signal at *δ* 83.1 (C-3′) in the HMBC spectrum, indicating fucose moieties linked at C-**3**′ of galactose. Meanwhile, the HMBC spectrum of **6** gave long range correlations between signals at *δ* 169.1 (C-1′′′) and *δ* 4.89 (H-4′′); *δ* 72.3 (C-1) and *δ* 4.34 (H-1′), indicating a caffeoyl residue at C-4′′, corresponding to the chemical shift (*δ* 4.89) of H-4'' with a downfield effect of the carbonyl group, and phenethyl group at C-1′ of galactose. Thus, compound **6** was established as 1′-*O*-β-(3,4-dihydroxyphenyl)-ethyl- [4′′-*O*-caffeoyl-( α-l-fucopyranosyl)]-(l-3′)-d-galactopyranoside.

### 2.2. Bioactivity

The isolated compounds showed strong antioxidant activity and highly decreased DPPH free radical levels, and their antioxidant activity were stronger than that of positive control ascorbic acid (Table 3). Compound **5** showed the highest antioxidant activity among all tested compounds, with an IC_50_ value of 0.12 µM. In addition, the antioxidant ability of compounds **3**, **5**, **6** was more than that of compounds **1**, **2**, **4**, suggesting that apart from phenolic hydroxy groups, the galactose group also plays an important role in scavenging DPPH free radicals.

**Table 3:** The scavenging activity of compounds **1**–**6** on DPPH free radicals.

Compound	IC_50_ (µM) ± SD
**1**	2.33 ± 0.06
**2**	1 ± 0.02
**3**	0.66 ± 0.04
**4**	1.15 ± 0.04
**5**	0.12 ± 0.03
**6**	0.24 ± 0.01
Ascorbic acid	3.08 ± 0.06

Apart from their antioxidant activity, their inhibitory effects on CYP3A4 enzyme were further studied. The data (Table 4) showed phenylpropanoid glycosides had inhibitory effects on cytochrome CYP3A4 enzyme. Compound **6** was the most active, with an IC_50_ value of 15.1 µM. As in the antioxidant assay, the inhibitory effect on CYP3A4 enzyme of compounds **3**, **5**, **6** was more than that of compounds **1**, **2**, **4**, suggesting that the galactose group plays an important role in the inhibitory activity on CYP3A4 enzyme.

**Table 4:** Inhibitory activity on CYP3A4 enzyme of compounds **1**–**6**.

Compound	IC_50_ (µM) ± SD
**1**	46.27 ± 1.53
**2**	43.3 ± 1.79
**3**	37.73 ± 2.11
**4**	91.7 ± 5.44
**5**	22.49 ± 1.65
**6**	15.1 ± 2.43
Ketoconazole	6.44 ± 0.56

## 3. Experimental

### 3.1. General

Optical rotations were determined with a Horiba SEPA-3000 high sensitivity polarimeter. UV spectra were measured on a Shimadzu UV-1600 visible spectrometer. IR spectra were recorded on a Shimadzu IR-8400 IR spectrophotometer. NMR spectra were obtained using a JEOL Delta 600 spectrometer in CD_3_OD with TMS as internal standard. Mass spectra were measured on a JEOL SX-102 mass spectrometer. Reversed-phase HPLC (5 µm, Waters) was performed. Silica gel (63-210 µm, Wako), ODS (63-212 µm, Wako) and Sephadex LH-20 (Sigma) were used for open column chromatography. TLC was performed on silica gel 60 F_254_ and RP-18 F_254_ (Merck).

### 3.2. Plant Material

Dried bark of *Tabebuia avellanedae* for the present investigation was kindly provided and taxonomically identified by Taheebo Japan Co., Ltd (Japan). A voucher specimen was deposited in a database at Kanazawa University Graduate School of Natural Science and Technology under registration numbers 2008-2010.

### 3.3. Extraction and Isolation

Dried bark of *Tabebuia avellanedae* (10 kg) was extracted with boiling water (30 L) three times. The water solutions were combined and concentrated *in vacuo*, and the residue (90 g) was suspended in H_2_O (1 L) and partitioned with EtOAc (1 L × 3) to yield EtOAc-soluble (14.5 g) and H_2_O-soluble (72.8 g) fractions, respectively. The EtOAc-soluble fraction (13 g) was chromatographed with a gradient solvent system (Hexane/EtOAc/MeOH) to give 15 fractions (300 mL). Fraction 13 (EtOAc/MeOH = 4/1, 3.5 g) was rechromatographed on ODS with gradient solvent (MeOH/H_2_O) to afford 18 subfractions (60 mL). Subfraction 6 (MeOH/H_2_O = 1/3, 76 mg) was subjected on ODS HPLC with 30% MeOH to yield 2 subfraction, subsequently, subfraction 1 (32 mg) was subjected on ODS HPLC with 26% CH_3_CN (containing 0.1% formic acid) to afford compound **1** (12.0 mg), **2** (7.5 mg), and **3** (8 mg), subfraction 2 (26 mg) was rechromatographed on ODS HPLC with 28% CH_3_CN (containing 0.1% formic acid) to yield **4** (10.2 mg), **5** (6.5 mg) and **6** (7.2 mg), respectively. For the ^1^H-NMR and ^13^C-NMR spectral data of compounds **1**, **3** and **4**, see Table 1.

*Compound* **2**. Amorphous powder, 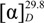 −128 (MeOH *c* 0.45); UV 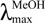 nm (log ε): 238 (4.40), 352 (4.24) nm; IR (KBr) cm^−1^, 3130, 3028, 2401, 2318,1753, 1720, 1697, 1548, 1506, 766; ^1^H-NMR and ^13^C-NMR spectra data see Table 1; HR-FABMS *m/z* 625.2126 [M+H]^+^ (calculated for C_29_H_37_O_15_ 625.2203).

*Compound* **5**. Amorphous powder, 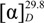 −105 ( MeOH *c* 0.1 ); UV 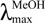 nm (log ε): 236 (4.39), 282 (4.33), 346 (4.37) nm; IR (KBr) cm^−1^, 3125, 1701, 1525, 1510, 1045; ^1^H-NMR and ^13^C-NMR spectral data see Table 2; HR-FABMS *m/z* 625.2135 [M+H]^+^ (calculated for C_29_H_37_O_15_ 625.2169). 

*Compound* **6**. Amorphous powder, 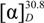 65 (MeOH *c* 0.1); UV 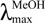 nm (log ε): 236 (4.31), 346 (4.29) nm; IR (KBr) cm^−1^, 3120, 1701, 1552, 1525, 1510; ^1^H-NMR and ^13^C-NMR spectral data see Table 2; HR-FABMS *m/z* 625.2144 [M+H]^+^ (calculated for C_29_H_37_O_15_ 625.2183).

### 3.4. Acid Hydrolysis of Compounds **2**, **5**, **6**

A solution of **2** (4.0 mg) in 1 M HCl (dioxane-H_2_O, 1:1; 2 mL) was heated at 95 °C for 2 h under an Ar atmosphere. After cooling, the reaction mixture was neutralized by passage through an Amberlite IRA-93ZU (Organo, Tokyo, Japan) column and then subjected to silica gel eluted with CHCl_3_-MeOH (9:1 to 1:1) to yield aglycone and a sugar fraction (1.5 mg). HPLC analysis of the sugar fraction under the following conditions showed the presence of d-glucose and l-fucose. Column: Capcell Pak NH_2_ UG80 (4.6 mm i.d. × 250 mm, Shiseido, Japan); detector: Shodex OR-2 (Showa-Denko, Japan); solvent, MeCN/H_2_O (17:3); flow rate, 0.9 mL/min; Identification of l-fucose and d-glucose present in the sugar fraction was carried out by comparison of their retention times and optical rotations with those of authentic samples. *t*_R_ (min): 12.51 (l-fucose, negative optical rotation), 18.55 (d-glucose, positive optical rotation).

Compound **5** (3 mg) was subjected to acid hydrolysis as described for **2** to give a sugar fraction (1.3 mg). HPLC analysis of the sugar fraction under the same conditions as in the case of that of **2** showed the presence of d-galactose and l-rhamnose, *t*_R_ (min): 13.41 (l-rhamnose, negative optical rotation); 18.23 (d-galactose, positive optical rotation).

Compound **6** (3 mg) was subjected to acid hydrolysis as described for **2** to give a sugar fraction (1.3 mg). HPLC analysis of the sugar fraction under the same conditions as in the case of that of **2** showed the presence of d-galactose and l-fucose, *t*_R_ (min): 12.65 (l-fucose, negative optical rotation); 18.34 (d-galactose, positive optical rotation).

### 3.5. Antioxidant Assay

Test samples (100 µL) at different concentrations in MeOH and DPPH (200 µL, 6 × 10^−5^ µM) in MeOH were added to 96-well microtiter plates. The plate was shaken for 1 min by a plate shaker, and incubated for 30 min at room temperature in the dark. After incubation, the absorbance was recorded at 517 nm. The tested samples at different concentrations without DPPH solution were used as blank control to eliminate the influence of the sample color. Ascorbic acid was used as positive control, and DPPH solution in MeOH served as a negative control. All tests were independently performed in triplicate. DPPH radical scavenging activity (%) = [1 − (Abs_S_ − Abs_B_)/Abs_C_] × 100%.

### 3.6. Inhibitory Activity on Cytochrome CYP3A4 Enzyme

Measurement of inhibitory activity on cytochrome CYP3A4 enzyme vivid substrate and fluorescent standards were reconstituted and a standard curve was prepared. 40 µL of test samples at different concentrations diluted in 1% DMSO, positive inhibition control and solvent were added to each 96-well plate, then was added 50 µL of Master pre-mix consisting of P450 BACULOSOMES in vivid CYP 450, Reaction Buffer and Regeneration System (consisting of glucose-6-phosphate and glucose-6-phosphate dehydrogenase), and the plate was mixed and incubated for 20 min to allow samples to interact with the CYP3A4 enzyme. The reaction was started by addition of 10 µL reaction starting liquid including vivid substrate, NADP and Reaction Buffer mixture, and the fluorescence was recorded using a LAS-3000 luminescent image analyzer at a wavelength of 460 nm after 30 min duration of reaction. The inhibition rate was calculated using the equation: % inhibition = [1 − relative fluorescence units (rfu) in the presence of the test sample or positive inhibition control/rfu in the absence of the test compound or positive inhibition control] × 100.

## 4. Conclusions

The study identified six phenylpropanoid glycosides from the water extract of Taheebo. Phenylpropanoid glycosides displayed strong anti-oxidant activity and moderate inhibitory activity on CYP3A4 enzyme. It is reported that oxidative stress is implicated in a wide array of human diseases, including cancer, neurodegenerative diseases, diabetes, inflammatory joint diseases, cardiovascular dysfunctions, as well as ageing, so the presence of strong anti-oxidant phenylpropanoid glycosides in Taheebo may be a good illustration supporting the many biological activities of Taheebo displays and its use in folk medicine to treat many diseases for thousands of years.
